# Quality of Pharmaceutical Advertisements in Medical Journals: A Systematic Review

**DOI:** 10.1371/journal.pone.0006350

**Published:** 2009-07-22

**Authors:** Noordin Othman, Agnes Vitry, Elizabeth E. Roughead

**Affiliations:** 1 Quality Use of Medicines and Pharmacy Research Centre, School of Pharmacy and Medical Sciences, University of South Australia, Adelaide, Australia; 2 Kulliyyah of Pharmacy, International Islamic University Malaysia, Jalan Istana, Bandar Indera Mahkota, Kuantan, Pahang, Malaysia; University of South Florida, United States of America

## Abstract

**Background:**

Journal advertising is one of the main sources of medicines information to doctors. Despite the availability of regulations and controls of drug promotion worldwide, information on medicines provided in journal advertising has been criticized in several studies for being of poor quality. However, no attempt has been made to systematically summarise this body of research. We designed this systematic review to assess all studies that have examined the quality of pharmaceutical advertisements for prescription products in medical and pharmacy journals.

**Methods and Findings:**

Studies were identified via searching electronic databases, web library, search engine and reviewing citations (1950 – February 2006). Only articles published in English and examined the quality of information included in pharmaceutical advertisements for prescription products in medical or pharmacy journals were included. For each eligible article, a researcher independently extracted the data on the study methodology and outcomes. The data were then reviewed by a second researcher. Any disagreements were resolved by consensus. The data were analysed descriptively. The final analysis included 24 articles. The studies reviewed advertisements from 26 countries. The number of journals surveyed in each study ranged from four to 24 journals. Several outcome measures were examined including references and claims provided in advertisements, availability of product information, adherence to codes or guidelines and presentation of risk results. The majority of studies employed a convenience-sampling method. Brand name, generic name and indications were usually provided. Journal articles were commonly cited to support pharmaceutical claims. Less than 67% of the claims were supported by a systematic review, a meta-analysis or a randomised control trial. Studies that assessed misleading claims had at least one advertisement with a misleading claim. Two studies found that less than 28% of claims were unambiguous clinical claims. Most advertisements with quantitative information provided risk results as relative risk reduction. Studies were conducted in 26 countries only and then the generalizability of the results is limited.

**Conclusions:**

Evidence from this review indicates that low quality of journal advertising is a global issue. As information provided in journal advertising has the potential to change doctors' prescribing behaviour, ongoing efforts to increase education about drug promotion are crucial. The results from our review suggest the need for a global pro-active and effective regulatory system to ensure that information provided in medical journal advertising is supporting the quality use of medicines.

## Introduction

Advertising in medical journals is one of the techniques used by pharmaceutical companies to promote their products to medical doctors. During the first four years of a new medicine on the market, pharmaceutical companies may gain approximately US $2.43 for each dollar spent on medical journal advertisements for a medicine [1]. The return on investment has been reported to increase to more than US $4 after that period [1].

Doctors use advertising in medical journals as one of the main sources of information for newly marketed drugs [2], [3], [4], [5]. Therefore, ideally, information provided in advertisements should be of high quality to support doctors to practice evidence-based medicine.

Internationally, two sets of guidelines have been developed for pharmaceutical advertising. In 1988, the World Health Organization (WHO) established the Ethical criteria for medicinal drug promotion [6]. These criteria constitute general principles for ethical standards that can be adapted by governments to national circumstances. The International Federation of Pharmaceutical Manufacturers Association (IFPMA) has adopted a Code of Pharmaceutical Marketing Practices, supplemented by member association and company codes, that sets standards for the ethical promotion of medicines [7]. It is a requirement of IFPMA membership that member associations adopt codes that meet local requirements but are consistent with, and as comprehensive as, the IFPMA Code. The IFPMA seeks to ensure that ethical promotional practices are established worldwide. These guidelines provide recommendations on the type and quality of information that should be included in journal advertisements. In most countries, regulation of the quality of advertisements in medical journals is a responsibility of government agencies [8] and/or the pharmaceutical industry [9]. Pharmaceutical industry codes of conduct most often complement the requirements set in legislation by developing standards and investigating alleged breaches [9].

Despite the availability of regulations and controls of drug promotion worldwide, pharmaceutical advertising in medical journals has been criticized for being of poor quality [10], [11], [12], [13], [14], [15], [16], [17], [18], [19], [20], [21], [22]. Several studies have assessed the quality of pharmaceutical advertisements and examined a range of different outcome measures [10], [11], [12], [14], [17], [18], [20], [22], [23], [24], [25], [26], [27], [28], [29], [30], [31], [32], [33] such as availability of product information [14], [29], [30], [31], type and truthfulness of marketing claims [20], [24], quality and availability of references provided to support the claims [19], [34], presentation of scientific results in terms of absolute or relative risk reductions [17], [20], quality of graphs [12] and overall compliance with the national regulations or guidelines [10], [11]. The overall results of these studies have never been synthesized. A systematic review would provide researchers and policy makers with information on the standards of pharmaceutical advertisements that may reflect the effectiveness of current guidelines and regulations.

We aimed to do a systematic review of all studies that have examined the quality of pharmaceutical advertisements for prescription products published in medical and pharmacy journals.

## Methods

### Selection Criteria

Studies were included in the review if they were published in English and examined the quality of information included in pharmaceutical advertisements for prescription products in medical or pharmacy journals. Studies were excluded from the review if they met one of the following conditions:

- only evaluated advertisements provided in pamphlets, brochures, leaflets and inserts. Unlike advertisements in medical journals, there is no repository of pamphlets, brochures and others which makes it very difficult to collect appropriate and representative samples for a study.
- assessed advertisements for both prescription and non-prescription medicines without separating the results,
- assessed advertisements for both prescription medicines and medical devices without separating the results,
- only assessed gender, metaphors or race issues in advertisements,
- assessed outcomes that were not related to the quality of medical information such as the use of pharmaco-economic terms and patterns of advertising.

### Search Strategy

We searched Medline (from 1950), International Pharmaceutical Abstracts (from 1970), Current Contents (from 1998), Scopus, Sociological Abstracts (from 1952), PsychInfo (1950) and Business Source Complete (from 1950). We also searched the Drug promotion database [35], Google Scholar, Web of Science (from 1993), and the Healthy Skepticism web library [36]. The first 100 results returned by each search from the Google Scholar were scanned for relevant articles.

We searched the databases for all studies published up to February 2006. The following combinations of search terms were used: “pharmaceutical”, “advertising or advertisements”, “promotion”, “codes of conduct”, “medical journal”, “marketing”,” journal”, “physicians” and “quality” and “information”. One researcher carried out the search and scanned the title and abstracts of studies identified from this search. A copy of all articles potentially eligible was retrieved and screened by the same reviewer for the inclusion criteria. All bibliographies of selected papers were screened for additional relevant articles.

### Data Extraction

Data extraction forms were developed to collect data on study design and study outcomes. For each eligible article, a researcher extracted the data. A second researcher then reviewed the data. All disagreements were resolved by consensus.

Information extracted on study design included: sampling methodology, total number of advertisements surveyed, total number of distinct advertisements surveyed, number of reviewers, consistency of reviewers, year of publication, period and country studied, type and number of journals evaluated.

The outcome measures examined included the availability of product information, the quality and availability of the references, the presentation of the risk results and the nature and quality of promotional claims.

### Availability of Product Information

We extracted the proportion of advertisements that included information on brand name, generic name, indications, side effects, dosage, interactions, precautions and contraindications, warnings and treatment of overdose.

### References

We examined information on references including the proportion of advertisements that used references, types of references provided to support marketing claims, quality of references, source of research funding of references and response from pharmaceutical companies to a requests for data on file.

### Claims

We extracted information on number and type of claims, number of misleading claims and proportions of advertisements compliant with codes or guidelines.

### Risk Results

We extracted proportions of advertisements mentioning relative risk reduction (RRR), absolute risk reduction (ARR) and number needed to treat (NNT).

### Data Analysis

As studies calculated results differently either on the basis of the total number of advertisements or on the total number of distinct advertisements (similar advertisements may be repeated in journals), we recalculated the results based on the total number of distinct advertisements when the raw data were available.

Data were entered using Microsoft Office Excel 2003. Descriptive statistics were produced for each outcome. A narrative synthesis method was used to enable us to analyse a large and diverse evidence base.

## Results

**Figure 1:**
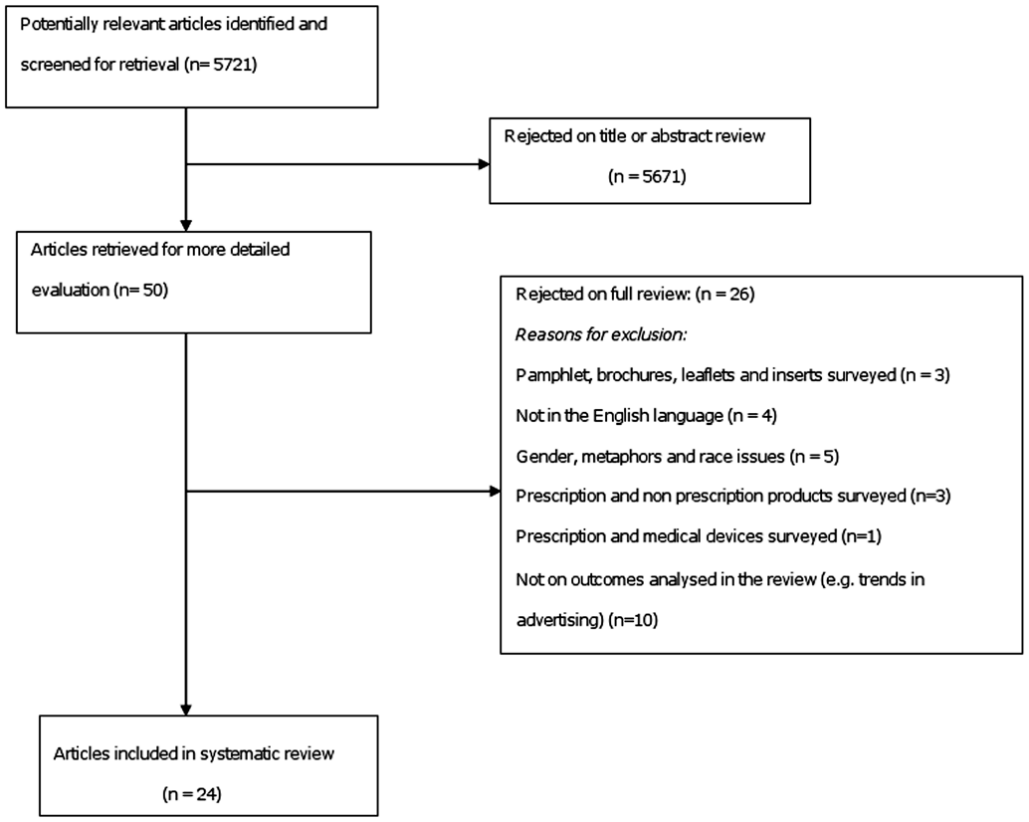
Literature Search and Study Selection.

Fifty articles were identified and 24 were included in the systematic review (Table 1) [10], [11], [14], [15], [17], [18], [19], [20], [21], [22], [24], [26], [27], [28], [29], [30], [31], [34], [37], [38], [39], [40], [41], [42]. Twenty-six studies [3], [13], [23], [25], [33], [43], [44], [45], [46], [47], [48], [49], [50], [51], [52], [53], [54], [55], [56], [57], [58], [59], [60], [61], [62], [63] were excluded after full review. The reasons for exclusion are detailed in the study flow diagram in Figure 1.

**Table 1:** General Characteristic of Studies.

Study (First author)	Country surveyed	Year of publication	Number of journal examined	Period studied	Number of advertisements	Number of distinct advertisements	Sampling method
Lexchin [24]	Canada	1999	5	1998	571	130	Convenience sample
Lankinen [17]	Finland	2004	4	2002	1036	245	Convenience sample
Carandang [10]	Australia	1994	4	1991 and 1992	Not reported	127	Convenience sample
Vlassov [31]	Russia	2001	5	1998	397	207	Convenience sample
Chirac [11]	French speaking African countries (West African and Maghreb)	1993	6	1990	1311	141	Convenience sample
Cooper [19]	The US	2005	10	1999	Not reported	438	Convenience sample

Study (First author)	Country surveyed	Year of publication	Number of Journal	Period studied	Total advertisements	Number of distinct advertisements	Sampling method
Gilad [37]	Israel	2005	24	2000	779	Not reported	Convenience sample
Gitanjali [38]	India and Britain	1997	2	1992 and 1993	203	Not reported	Convenience sample
Lal [39]	India	1992	19	1990–1991	903	Not reported	Convenience sample
Stimson [34]	The UK	1976	19	1974	1104	89	Convenience sample
Moulds [28]	Australia	1987	5 (results only given for 3)	1986	274	Not reported	Convenience sample

Study (First author)	Country surveyed	Year of publication	Number of Journal	Period studied	Total advertisements	Number of distinct advertisements	Sampling method
Herxheimer [14]	18 countries. (Finland, Norway, Sweden, Denmark, Spain, France, Italy, Ireland, UK, Switzerland, Turkey, Indonesia, Nepal, Pakistan, Sri Lanka, Tanzania, Zimbabwe and Brazil)	1993	23	1987 to 1988	6710	No reported	Convenience sample
Mastroianni [30]	Brazil	2003	4	1985 to 2001	Not reported	199	Convenience sample
Villanueva [22]	Spain	2003	6	1997	954	287	Convenience sample

Study (First author)	Country surveyed	Year of publication	Number of Journal	Period studied	Total advertisements	Number of distinct advertisements	Sampling method
Stimson [42]	The UK	1975	27	1974	3895	591	Convenience sample
Mould [26]	Australia	1986	5	1985	Not reported	138	Convenience sample
Mindell [40]	UK	1997	1	1997	Not reported	46	Convenience sample
Wilkes [15]	The US	1992	10	1990	143	109	Convenience sample
Lal. [29]	India, UK and the US	1997	Not reported	From July 1994 to June 1995	Not reported	762	Random sample

Study (First author)	Country surveyed	Year of publication	Number of Journal	Period studied	Total advertisements	Number of distinct advertisements	Sampling method
Loke [20]	Australia	2002	6	Oct to Dec 2000	1000	174	Convenience sample
Gutknecht [21]	The US	2001	4	1999	Not reported	187	Convenience sample
Lexchin [18]	Canada	1994	1	1990	Not reported	22	Convenience sample
Moulds [27]	Australia	1989	1	1988	56	Not reported	Convenience sample
Smart [41]	The UK	1997	1	1996	Not reported	81	Convenience sample

### Characteristics of Studies Included

The studies reviewed advertisements from 26 countries predominantly the United Kingdom (UK) (7/24, 29%), Australia (5/24, 21%) and the United States (US) (4/24, 17%) (Table 1). Most studies (19/24, 79%) assessed the quality of advertisements in developed countries. All studies were cross-sectional studies.

All studies were published between 1975 and February 2006. Five studies (5/24, 21%) were published between 1975 and 1990, eleven (11/24, 46%) between 1990 and 2000 and eight (8/24, 33%) between 2000 and 2005.

The studies were conducted with three main objectives. Four studies (4/24, 17%) compared the quality of advertisements in different countries. Sixteen studies (16/24, 67%) assessed the quality of advertisements in a single country. Five studies (5/24, 21%) compared advertisements published at different times [10], [26], [27], [28], [30]. Four of the five studies conducted in Australia used the same methodology and three of these were done by the same researchers enabling comparison overtime [10], [26], [27], [28].

Twenty-three studies surveyed advertisements in medical journals and one study assessed advertisements in medical and paramedical journals. The number of journals surveyed in each study ranged from one to 24 journals. Eighteen studies (18/24, 75%) provided information on the total number of distinct advertisements analyzed. Nine studies (9/24, 38%) reported both the total number advertisements and number of distinct advertisements examined in their studies. The total number of advertisements evaluated ranged from 56 to 6710 (median = 903) and the number of distinct advertisements ranged from 22 to 762 (median = 158).

Several types of outcome measures were examined (Table 2). Sixteen studies (16/24, 67%) assessed references provided in advertisements, nine studies (9/24, 38%) examined availability of product information and nine studies (9/24, 38%) assessed claims provided in advertisements. Four studies (4/24, 17%) evaluated presentation of risk results and seven studies (29%) assessed adherence to codes or guidelines.

**Table 2:** Outcome Measures.

Outcome measure(s)	Study (First author)	Number of studies
**Product information**		
Availability	Carandang [10], Vlassov [31], Chirac [11], Gitanjali [38], Lal [39], Mastroianni [30], Herxheimer [14], Lal [29], Stimson [42].	9
**References**		
Availability of references	Lankinen [17], Carandang [10], Vlassov [31], Cooper [19], Gilad [37], Lal [39], Stimson [34],Smart [41], Herxheimer [14], Mastroianni [30], Villanueva [22], Mindell [40], Wilkes [15], Lal [29]	14
Type of references	Carandang [10], Cooper [19], Stimson [34], Smart [41], Villanueva [22], Mindell [40], Lexchin [18], Herxheimer [14],	8
Quality of references	Lankinen [17], Villanueva [22], Loke [20], Mindell [40], Smart [41], Lexchin [18], Stimson [34]	7
Type of outcomes measure in references	Villanueva [22]	1
Availability of information on sponsorship	Stimson [34], Villanueva [22], Cooper [19]	3
**Claims**		
Number of marketing claims with reference	Lankinen [17], Villanueva [22]	2
Number of advertisements with medical claims	Villanueva [22]	1
Type of claims	Lankinen [17], Loke [20],	2
Validity of claims	Gitanjali [38], Moulds [28], Villanueva [22], Moulds [26], Herxheimer [14], Wilkes [15], Moulds [27]	7
**Risks results**		
Methods of presentation of risk results	Lexchin [24], Lankinen [17], Loke [20], Gutknecht [21]	4
**Adherence to codes or guidelines**		
Compliance with codes or guidelines.	Carandang [10], Chirac [11], Moulds [28], Mastroianni. [30], Moulds.[26], Wilkes [15], Moulds [27]	7
**Response provided by pharmaceutical companies upon a request for references**		
Response upon a request for references	Stimson [34], Lexchin [18], Cooper [19], Lal [39]	4

### Study Quality

Seventeen studies (17/24, 71%) provided data on the number of assessors. Three studies (3/24,13%) used one assessor, eight studies (33.3%) used two assessors and five studies (2/24, 21%) used between three to five assessors. One multi-country study had different assessors per country [14] who may have applied different standards. Five studies (5/24, 21%) provided information on the consistency of assessors. Of the three studies (3/24, 12%) that reported kappa scores, good and excellent agreement was noted.

Twenty-three studies (23/24, 96%) used a convenience-sampling method. Six studies (6/24, 25%) selected journals based on readership [14], [15], [18], [22], [24], [31]. One study (1/24, 4%) used random sampling but no information was given on how the randomization was conducted [29].

### Availability of Product Information

Six of the eight studies (6/24, 25%) that recorded information on generic name found that generic names were mentioned in 83 to 100% of advertisements (median = 90%) (Table 3). A UK study in 1975 [42] and a Russian study in 2001 [31] found lower rates, 43% and 39% respectively.

**Table 3:** Availability of Product Information.

Study (First author)	Results calculated based on	Brand name n (%)	Generic name n (%)	Indications n (%)	Contraindications n (%)	Interactions n (%)
Carandang 1994 [10]	Distinct advertisements	Not reported	115/127 (91)	Not reported	Not reported	Not reported
Chirac 1993 [11]	Distinct advertisements	Not reported	125/141 (89)	136/141 (96)	97/141 (69)	Not reported
Mastroianni 2003 [30]*	Distinct advertisements	Not reported	34/39 (87), 29/31 (90)	37/39 (95), 31/31 (100)	20/39 (51), 20/31 (64)	20/39 (51), 21/31 (68)
		Not reported	17/19 (89), 18/19 (95)	18/19 (95), 19/19 (100)	10/19 (53), 12/19 (63)	9/19 (47), 11/19 (58)
		Not reported	51/60 (85), 34/34 (100)	57/60 (95), 33/34 (97)	22/60 (37), 25/34 (74)	22/60 (37), 25/34 (73)
Stimson 1975 [42]	Distinct advertisements.	544/591 (92)	255/591 (43)	Not reported	25/591 (4)	Not reported
Vlassov 2001 [31]	Number of placements.	A few advertisements	154/397(39)	177/397 (45)	42/397 (11)	21/397 (5)
**Study (First author)**	**Results calculated based on**	**Brand name n (%)**	**Generic name n (%)**	**Indications n (%)**	**Contraindicationsn (%)**	**Interactions n (%)**
Lal 1992 [39]	No detail information was given	(100)	(84)	(80)	(10)	(3)
Herxheimer 1993 [14] (18 countries)	No detail information was given	Not reported	Not reported	(94),(86),(90),(94), (91),(97),(34),(93), (97),(80),(91),(94), (81),(77), (84), (40),(97),(98)	(1),(67),(21),(88), (88),(73),(35),(35), (93),(8),(33),(18), (-), (35), (22), (20),(24), (43)	Not reported
Lal 1997 [29]	No detail information was given	(100),(100), (100)	(89), (98), (99)	(80), (92), (97)	(13),(82), (91)	(8),(63), (38)

Study (First author)	Dosage regimen n (%)	Drug overdose n (%)	Side effects n (%)	Warnings n (%)	Precautions n (%)
Carandang 1994[10]	Not reported	Not reported	Not reported	Not reported	Not reported
Chirac 1993 [11]	123/141 (87)	Not reported	99/141 (70)	Not reported	Not reported
Mastroianni 2003 [30]*	26/39 (67), 26/31 (84)	Not reported	20/39(51) 20/31 (64)	20/39 (51) 22/31 (71)	Not reported
	19/19(100), 17/19 (90)	Not reported	10/19(53), 10/19 (53)	10/19 (53), 12/19 (63)	Not reported
	35/60 (58), 28/34 (82)	Not reported	22/60 (37), 25/34 (74)	21/60 (35), 26/34 (76)	Not reported
Stimson 1975 [42]	84/591 (14)	Not reported	23/591 (4)	Not reported	18/591 (3)
Vlassov 2001 [31]	Not reported	Not reported	Not reported	44/397 (11)	Not reported
Gitanjali 1997[38]	Not reported	Not reported	Not reported	Not reported	Not reported

Study (First author)	Dosage regimen n (%)	Drug overdose n (%)	Side effects n (%)	Warnings n (%)	Precautions n (%)
Lal 1992 [39]	(73)	Not reported	(9)	(10)	(10)
Herxheimer 1993 [14] (18 countries)	Not reported	Not reported	(1), (71), (31), (92), (88), (81), (35), (35), (94), (6), (39), (18), Not Known, (32), (24), (16), (27), (57)	(18),(65), (34), (48), (80), (83), (35), (35), (95), (7), (38), (17), (-), (31), (34), (24), (-), (40)	In this study warning and precaution is reported in the same group
Lal 1997 [29]	(31),(77),(97)	(2),(44), (23)	(14), (83), (82)	(9), (77), (60)	(12), (83), (80)

Approved indications were mentioned in more than 70% (median = 94% ) of advertisements in five studies [11], [14], [29], [30], [39]. Lower rates were observed in the Russian study (45%) [31] and in some countries (Italy, 34%, Tanzania, 40%) in a multi-country study [14]. Six studies (6/24, 25%) that examined information on side effects reported mixed results [11], [14], [29], [30], [39], [42]. Five studies (5/24, 21%) reported low rates of information, around 14% or less (median = 6%) in India [29], [39], Finland [14], Switzerland [14] and in an 1975 UK study [42]. Two studies (2/24, 8%) published in 1993 and 1997 reported rates over 80% (median = 83%), namely in the US [29], Denmark [14], Spain [14], France [14] and the UK [14], [29].

Studies that examined information on contraindications [11], [14], [29], [30], [31], [39], [42], warnings [14], [29], [30], [31], [39], precautions [14], [29], [39], [42] reported variable findings. Six studies (6/24, 25%) that reported information on contraindications found that contraindications were mentioned in less than 74% (median = 35%). Two studies (2/24, 8%) reported that contraindications were mentioned in 82 to 93% (median = 88) of advertisements in Denmark [14], Spain [14], the UK [14], [29] and the US [29]. Information on warnings was mentioned in less than 77% (median = 35%) of advertisements in five studies (5/24, 21%). One study reported that warnings were mentioned in 80 to 95% (median = 83%) of advertisements namely in Spain [14], France [14], and the UK [14]. Four studies that reported information on precautions found that precautions were mentioned in less than 65% (median = 32%). Two studies (2/24, 8%) reported that precautions were mentioned in 80 to 95% (median = 83) of advertisements in Spain [14], France [14], the UK [14], [29] and the US [29].

Five studies (5/24, 21%) reported information on dosage (range: 14–100%, median = 80%) [11], [29], [30], [39], [42]. High results were noted in advertisements that appeared in the UK from July 1994 to June 1995 (97%) [29] and in West African and Maghreb in 1990 (87%) [11]. Variable findings were noted before and after the implementation of regulations on advertisements in a study conducted in Brazil in 2003 (range = 58–100%, median = 83% ) [30]. Two Indian studies [29], [39] conducted in different years revealed different results [38].The study that was conducted in 1997 [29] found much lower (31%) information on dosage compared to the earlier study in 1992 (73%) [39].

### References

References were provided in more than half of the advertisements (range 51–100%, median = 65%) in all studies that evaluated advertisements in developed countries [15], [17], [19], [29], [34], [37], [40], [41] except a study published in Spain (13%) [22] (Table 4). References were more rarely provided (range 2–59%, median = 23%) in developing countries [29], [30], [31], [39]. Three studies (3/24, 12%) [17], [20], [22] found that between 18 to 37% (median = 32%) of references supporting claims were irretrievable.

**Table 4:** Availability of References.

Study (First author)	Number of advertisements with references n (%)
Mindell 1997 [40]	31/46 (67)
Lankinen 2004 [17]	245/245 (100)
Cooper 2005 [19]	312/438 (71)
Stimson 1976 [34]	89/89 (100)
Smart 1997 [41]	41/81 (51)
Mastroianni 2003 [30] *	6/39 (15), 7/31 (23)
	8/19 (42), 9/19 (47)
	33/60 (55), 20/34 (59)
Villanueva 2003 [22]	38/287 (13)
Wilkes 1992 [15]	69/109 (63)

Study (First author)	Number of advertisements with references n (%)
Vlassov 2001 [31]	8/397 (2)
Gilad 2005 [37] antibiotics/other drugs	(56) and (52)
Lal 1992 [39]	(5)
Herxheimer 1993 [14]	(20)
Lal 1997 [29]	(19), (68), (51)

* Advertisements published before and after 3 regulation were established.

### Type of References

Eight studies (8/24, 33%) assessed the type of references provided in pharmaceutical advertisements. Overall, the references most commonly cited were journal articles (range = 55 to 90%, median = 73%). Other types of evidence were data on file (range = 15–19%, median = 17%) [10], [14], [19], meeting abstract and presentations (range = 5–23%, median = 15%) [19], [22], [40], books or monographs (range = 5–18%, median = 8%), marketing reports (5%) [19], prescribing information (range = 6–20%, median = 13% ) [19], [40], government documents (4%) [19], and other evidence (1%).

### References - Source of Research Funding

Three studies (3/24, 12%) examined the funding of studies used in references (range = 39–58%, median = 40%) [19], [22], [34]. A study that was conducted in the US determined that the majority (58%) of the original research cited in the pharmaceutical advertisements was sponsored by or had an author affiliated with the product's manufacturer [19]. A Spanish study [22] found that 41 studies (40%) had been financed by the pharmaceutical industry. Similar findings were noted in a UK study of which 39% of references were sponsored by the industry [34].

### Response to Request for Data on File

Three studies (3/24, 12%) investigated how companies responded to request for data on file [18], [19], [39]. The response rates were 42%[19], 37% [39] and 60% [18].

### Quality of References

Of seven studies (7/24, 29%) [17], [18], [20], [22], [34], [40], [41] that examined the quality of references, four studies (4/24, 17%) [17], [20], [22], [41] assessed the level of evidence of the references cited to support marketing claims (Table 5). One to twelve percent of references (median = 2%) were supported by a systematic review or meta-analysis. More randomised control trials were cited in a Spanish study (67%) compared to studies published in the UK (30%), Finland (9%) and Australia (35%).

**Table 5:** Level of evidence.

Study (First author)	Level of evidence n (%)		
	Systematic review or Meta analysis	Randomized control trial	Other evidence
Smart et al, 1997 [41]	2/139 (1)	41/139 (30)	96/139 (69)
Lankinen et al, 2004 [17]	9/381 (2)	33/381 (9)	135/381 (36)
Loke et al, 2002 [20]	99/855 (12)	297/855 (35)	75/855 (9)
Villanueva et al, 2003 [22]	-	84/125 (67)	18/125 (14)

Three studies (3/24, 12%) examined other aspects of the quality of the references [18], [34], [40]. A UK study [40] found that only two fifths of advertisements cited were published, peer reviewed references. A Canadian study found that the mean methodological quality score (58%, 95% CI 51%–65%) and the mean relevance score (76%, 95% CI 72%–80%) of the references were significantly lower than the acceptable score of 80% (p<0.05) [18].The poor rating for methodological quality was primarily because of the citation of references to low-quality review articles and “other” sources [18]. A UK study [34] assessed whether the claims were supported by adequate references. Of 49 references cited to substantiate the claims, 14 (29%) were judged adequate on the basis of predetermined criteria including presence of adequate controls, randomisation of treatments, objective assessment and statistical analysis of results.

### Type of Claims

One study in Australia (2002) [20] and one in Finland (2004) [17] (2/24, 8%) used the same system to classify the claims provided in advertisements. Nine to 28% of the claims were about an unambiguous clinical outcome, 29 to 37% provided a vague clinical outcome, 20 to 31% an emotive or immeasurable outcome and 23% a non-clinical outcome.

### Validity of Claims

Seven (7/24, 29%) studies examined the validity of promotional claims [10], [14], [15], [26], [27], [28], [38]. Four Australian studies in 1986 [26],1987 [28],1989 [27] and 1994 [10] examined the proportion of misleading claims in Australian advertisements. Thirty-one percent of advertisements reviewed in 1986 and 1987 were judged to be misleading and 16% in 1989. A third study in 1994 with the same methods but a different principal investigator classified 8% of advertisements as misleading [10].

In a study [38] conducted in India, ten randomly selected advertisements from the Indian edition of British Medical Journal were sent to three experts. They found that all the advertisements were misleading or made unsubstantiated claims [38]. One multi-country study with a different evaluator in each country [14] found that relatively few (no detailed information provided) advertisements provided misleading information except in Brazil (25%), Finland (50%), Italy (30%) and Pakistan (38%). However, no definition of misleading information was given [14].

In 1992, an American study [15] reviewed 109 advertisements published in 10 medical journals and noted that headlines were found to mislead the reader about efficacy in 32% of advertisements. In 44% of cases, reviewers felt that the advertisement would lead to improper prescribing if the physician had no other information about the drug other than the advertisement [15].

A Spanish study [22] found that 44% of claims with citations were not supported by the reference, most frequently because it recommended the drug for a patient group other than that assessed in the study.

### Presentation of Risk Results

Four studies (4/24, 17%) reported information on how benefit and harm were presented in advertisements that reported changes in clinical outcomes (Table 6) [17], [20], [21], [24]. Between 7 and 22% (median = 7%) of advertisements provided information on risk results [20], [24]. In a Canadian study [24], half of the 22 advertisements that reported changes in clinical outcomes reported the RRR, none reported the ARR or NNT, but 41% provided data that would enable readers to calculate those figures if they knew how. In an Australian study [20], none of the claims explicitly reporting quantitative outcomes provided ARR or NNT. In two other studies, none of the advertisements provided NNTs [17], [21].

**Table 6:** Risk Results' Information.

Study (First author)	Advertisements with n (%)			
	Relative risk reduction	Absolute risk reduction	Number needed to treat	Original data permitting calculation by readers
Lexchin 1999 [24]	29/130 (22)	0 (0)	0 (0)	9/130 (7)
Lankinen 2004 [17]	No reported	1/245 (0.4)	0/245 (0)	Not reported
Loke 2002 [20]	13/174 (7)	0 (0)	0 (0)	2/174 (1)
Gutknecht 2001 [21]	Not reported	Not reported	0 (0)	Not reported

## Discussion

We found that pharmaceutical advertisements in medical journals usually provided brand and generic name and indication. Other essential information required for rational prescribing including contraindications, interactions, side effects, warnings and precautions were less commonly provided. The majority of references cited to support pharmaceutical claims were journal articles. However, less than two-third of the claims were supported by a systematic review or a meta-analysis (110/1375, 8%) and randomised control trial (455/1500, 30%). About half of references were sponsored or had researchers affiliated with pharmaceutical companies. Pharmaceutical companies often did not provide data on file when requested. Variable rates regarding misleading claims were noted. Only 28% or less of claims were unambiguous clinical claims. When presented, most advertisements provided information on risk results as RRR not as ARR and NNT.

Provision of balanced drug information is a necessary element in the promotion of the appropriate use of medicines. Doctors need to be informed about the benefits but also the risks of drugs. However, pharmaceutical companies' efforts in providing balanced information are debatable [13], [64]. A US congressional inquiry reported that from August 1997 to August 2002, the Food and Drug Administration (FDA) issued 88 letters accusing drug companies of advertising violations mainly because pharmaceutical companies overstated the effectiveness of their products [65]. Similarly, this review found that the negative effects of a drug, which may discourage use of that drug, less commonly appeared in advertisements. The IFPMA code serves as a model for individual country marketing codes. While it states that promotional information should be balanced, it appears that the concept of “balanced information” is not clear [7]. There appears to be no appropriate standard of balanced information. Therefore, clear definition on balanced information appearing in pharmaceutical advertisements should be determined. Future policies and regulations on journal advertising need to take account of this imbalance in information.

This review noted that references used to support pharmaceutical claims were often of low quality. The inappropriate use of references in journal advertising suggests that the availability of references does not always guarantee the quality of claims. Furthermore, current requirements of the IFPMA code of conduct on the use of references to support promotional claims is vague and open to abuse [7]. This review suggests that the IFPMA should strengthen its code by providing explicit requirement on scientific evidence that may facilitate the selection of appropriate references to support claims in journal advertising.

Our review noted that all studies that assessed misleading claims had at least one advertisement with a misleading claim. The results call into question the commitment of pharmaceutical companies to educate doctors about their products in order to establish a clear understanding of the appropriate use of prescription medicines [7]. This review also highlights the limitation of the IFPMA code which does not have a clear procedure to correct misleading claims in journal advertising [7]. Furthermore, assessment of the validity of promotional claims is difficult, this review lend support to calls for increased education about drug promotion [66].

This review found that when presenting quantitative results, journal advertising often provided information on risk results only as RRR. As incomplete presentation of quantitative data may influence doctors' prescribing behaviour [67], this review call into question the adequacy of the IFPMA code of conduct. According to section 4 (2) of the code: *“Promotional information should be clear, legible, accurate, balanced, fair, objective and sufficiently complete to enable the recipient to form his or her own opinion of the therapeutic value of the pharmaceutical product concerned. Promotional information should be based on an up-to-date evaluation of all relevant evidence and reflect that evidence clearly. It should not mislead by distortion, exaggeration, undue emphasis, omission or in any other way. Every effort should be made to avoid ambiguity”.* The code does not provide any detailed requirements on how quantitative results should be presented. This limitation highlights the need for IFPMA to amend its code with regards to the presentation of statistical information in journal advertising in order to support the quality use of medicines

Information on medicines is essential to help doctors ensure the optimal use of medicines. However, studies show that doctors who use journal advertisements as a source of information may prescribe less appropriately [68], [69]. In addition, reliance on journal advertising for information is associated with increased costs of prescribing [70], [71]. Even doctors who think that they obtain their knowledge from the scientific literature can be influenced by promotional sources without being aware of it [72]. As information provided in journal advertising has the potential to change doctors' prescribing behaviour, our review indicates that ongoing efforts including complaints and recommendations by researchers, health professionals and policy makers to improve the quality of advertisements in medical journals are crucial.

The Ethical Criteria for Medicinal Drug Promotion developed by the World Health Organization (WHO) [6] recommend a minimum set of medicines information for journal advertising. However, this review found that safety information was still missing in studies undertaken after the publication of the WHO Ethical Criteria. Since 1988, the WHO has not reviewed the ethical criteria concerning advertising in medical journals [6]. Since the criteria lay the foundation for behaviour concerning the pharmaceutical promotion, it may be necessary for WHO to be proactive in updating the requirements for these activities.

Journal advertising is typically governed through self-regulatory codes administered by industry associations. In most countries, the recognition of breaches is based on a complaints mechanism. Complaints of violations can only be made after advertisements have already been circulated. The current system is limited by retrospective detection of code breaches and has no prevention focus. Furthermore, there is evidence that many violations of marketing codes go unreported [73], [74] and only a small portion of promotional materials voluntarily submitted for comment before submission are reviewed [75]. The poor quality of information found in this review suggests that the current system may be unable to regulate journal advertisements effectively. This limitation highlights the need for governments and pharmaceutical industry to be jointly responsible for regulating journal advertising. Governments may need to take more proactive action such as engaging independent experts to help in designing regulation for journal advertising where self regulatory codes are limited. In addition to that, effective regulatory system may complement pharmaceutical litigation to ensure accuracy and reliability of information in journal advertising [76].

Most medical journals rely on advertising for part of their revenue. Dependence on revenue from the industry may minimize the independence of the medical journals [77], [78]. A survey in North American survey found that 21% of journals editors claimed that they did not review advertisements provided by the industry before their appearance in the journals [79]. The low quality of information provided in journal advertising noted by this review highlight the need of journal editors and publishers to consider regulatory controls for advertising in their publications. Introduction of journal own codes is expensive. Journals obviously need to have independent financial resources to remove the conflict of interest with pharmaceutical companies. The Public Library of Science (PLoS) [80], a non-profit scientific and medical publisher has provided a good model which can be copied by other journals. Sources of revenue for PLoS includes donations from individual, paid individual memberships, support from foundations, from institutional memberships, and from asking research funders to pay a publication charge for accepted research papers. In addition to that, medical journals' financial resources could be relied on the advertising of products other than those supplied by pharmaceutical companies [77].

Our review found that the low quality of journal advertising was a global issue. Poor quality advertising has been observed in developing countries and post-Soviet Russia where controls might be weak and limited as well as in developed countries which have stricter regulations [81]. IFPMA states that the industry has an obligation and responsibility to establish a clear understanding of the appropriate use of prescription medicines. Based on the results of this review, stronger enforcement mechanisms would appear necessary to encourage pharmaceutical companies to provide reliable information which is essential for the rational prescribing of promoted products as recommended by the code. This is particularly the case in developing countries and post-Soviet Russia where independent sources of information on medicines are limited and where doctors rely on industry for most medicine information [82], [83].

This systematic review provides the current body of evidence on the quality of advertising in medical journals which will assist researchers in designing future studies. However, the variability in outcomes utilised in assessing the quality of information in the studies made collation of results difficult. There appears to be no consensus among researchers on the most appropriate outcomes. Most studies assessed references, availability of product information and adherence to codes or guidelines as indicators for information quality. Pharmaceutical companies may provide advertisements adhering to guidelines and with complete information supported by strong research based evidence. However, this does not mean that the advertisements are supporting rational prescribing. There is a need for developing more appropriate indicators to assess the quality of information in advertisements. This effort will minimize the heterogeneity of data and will allow direct comparison between studies.

### Limitations

This review was limited to studies that had been published in English language. Excluding studies in other languages may have led to the omission of some studies that provide evidence about the quality of information in journal advertising.

No attempt was made to define what was meant by quality of information. Rather than entering into discussion regarding the definition of the quality, we decided to define it based on presence or absence of information, availability and level of evidence of references, type and number of misleading claim and proportion of advertisements compliant with code or guidelines and the presentation of risk results.

Only one of the 24 studies included in this review selected advertisements randomly and the report of that study did not specify the random selection procedure. Also the countries studied are not representative of all countries. Consequently extrapolation of the average findings of this review to the average for all advertisements around the world may not be accurate. The number of studies is too small and their methods and quality are too variable to allow confident overall conclusions about changes over time or differences between countries.

### Future Work

This review has noted several outcomes measures that have not been adequately investigated in research during the review period. Firstly, only one study assessed whether claims were supported by references [22]. Additional research on the use the references to support claims in journal advertising is needed. Secondly, the majority of studies that examined misleading claims were not well reported [10], [14], [26], [27], [28], [38]. Although it is difficult to judge misleading claims, this review demonstrates the need for development of a widely accepted definition of a misleading claim and development of well described methods that can be used in different countries and years to enable comparisons. Thirdly, all multinational studies that measured content of journal advertising were published before 1998. Since then, codes of conducts and regulations on pharmaceutical advertising have been updated. It will be useful to conduct a comparative international study to provide recent comparative data on journal advertising. The study should be conducted to compare the effects of different regulatory frameworks. The study would provide policy makers with recent evidence of the strengths and weaknesses of different systems. This information is crucial for improving standards and regulations for pharmaceutical promotion.

Most pharmaceutical markets are dominated by international companies. These companies have their own marketing standards which are often based on the standards set forth in the IFPMA code of conduct [84], [85]. According to the codes that are publicly available promotional materials should encourage the appropriate use of medicines by presenting information accurately, without exaggeration and must follow all relevant local laws and companies policies and procedures [84], [85]. This review noted that no independent study has been conducted to evaluate whether companies are implementing their codes in a uniform way across countries. It would be useful to conduct a study to compare how advertisements for the same medicines are presented in different countries. The study would provide the first data pertaining the adherence of pharmaceutical companies to their own ethical codes and local standards in the provision of medicines information in journal advertising for international marketing communications.

Journal advertising is one among various promotional practices. However, it has been reported that pharmaceutical companies are cutting back print media to promote medicines and increase their promotional activities on internet marketing [86], [87] and continuing medical education [88], [89]. Therefore it would be beneficial to conduct a review to examine the quality of medicines information in internet marketing and continuing medical education.

### Conclusion

Globally, pharmaceutical advertising in medical journals often provides poor quality information. The impact of this problem on doctors' prescribing behaviour might be even greater in developing countries and post-Soviet Russia where access to industry-free medicine information is limited. The results from our review suggest the need for a global pro-active and effective regulatory system to ensure that information provided in medical journal advertising is supporting the quality use of medicines.
